# Time-Series Forecasting of Hemodialysis Population in the State of Qatar by 2030

**DOI:** 10.5339/qmj.2023.6

**Published:** 2023-02-20

**Authors:** Abdullah Hamad, Anas Mefleh Al Halabi, Hafedh Ghazouani, Elmukhtar M. Habas, Abdelsalam Mohamed Borham, Sahar Mohamed Ismail, Hassan Ali Al-Malki, Mohamad M. Alkadi

**Affiliations:** ^1^Department of Medicine, Division of Nephrology, Hamad Medical Corporation, Doha, Qatar. E-mail: ahamad9@hamad.qa; ORCID: (0000-0003-4677-7686); ^2^Department of Quality and Patient Safety, Hamad Medical Corporation, Doha, Qatar.

**Keywords:** Forecasting, Prediction, Dialysis, Hemodialysis, Chronic Kidney Disease, Qatar, Times series, Prevalence. Model. Trends

## Abstract

Background: There are few statistics on dialysis-dependent individuals with end-stage kidney disease (ESKD) in Qatar. Having access to this information can aid in better understanding the dialysis development model, aiding higher-level services in future planning. In order to give data for creating preventive efforts, we thus propose a time-series with a definitive endogenous model to predict ESKD patients requiring dialysis.

Methods: In this study, we used four mathematical equations linear, exponential, logarithmic decimal, and polynomial regression, to make predictions using historical data from 2012 to 2021. These equations were evaluated based on time-series analysis, and their prediction performance was assessed using the mean absolute percentage error (MAPE), coefficient of determination (R^2^), and mean absolute deviation (MAD). Because it remained largely steady for the population at risk of ESKD in this investigation, we did not consider the population growth factor to be changeable. (FIFA World Cup 2022 preparation workforce associated growth was in healthy and young workers that did not influence ESKD prevalence).

Result: The polynomial has a high R^2^ of 0.99 and is consequently the best match for the prevalence dialysis data, according to numerical findings. Thus, the MAPE is 2.28, and the MAD is 9.87%, revealing a small prediction error with good accuracy and variability. The polynomial algorithm is the simplest and best-calculated projection model, according to these results. The number of dialysis patients in Qatar is anticipated to increase to 1037 (95% CI, 974–1126) in 2022, 1245 (95% CI, 911–1518) in 2025, and 1611 (95% CI, 1378–1954) in 2030, with a 5.67% average yearly percentage change between 2022 and 2030.

Conclusion: Our research offers straightforward and precise mathematical models for predicting the number of patients in Qatar who will require dialysis in the future. We discovered that the polynomial technique outperformed other methods. Future planning for the need for dialysis services can benefit from this forecasting.

## Introduction

End-stage kidney disease (ESKD) is a growing global healthcare concern.^1^ The number of patients in Qatar with ESKD requiring hemodialysis (HD) and peritoneal dialysis (PD) has amassed over the last two decades by more than four and eight times, respectively.^2-4^ This growth coincides in part with a roughly four-fold increase in Qatar's population.^5,6^ Other variables include decreased mortality in the ESKD population and increased occurrence of risk factors for ESKD (diabetes mellitus, hypertension, obesity, etc.).^7-9^ In Qatar, only several government healthcare facilities run by the Hamad Medical Corporation have ever offered dialysis services. There were 250 PD patients (22%) and 900 HD patients (78%), using more than 80% of the available HD stations across the country as of January 1, 2022.^10-12^

There are data on worldwide epidemiology, temporal trends, and predictors of ESKD requiring dialysis in the future in Qatar. Forecasts can also be a useful tool for improving and promoting healthcare provided they are accurate enough to allow for prompt corrective action, assisting healthcare decision-makers to allocate resources and plan financial strategies.

Here, we seek to improve and promote the ESKD program by utilizing time-series analysis to forecast the prevalence of ESKD patients needing dialysis in Qatar from 2022 to 2030.

In the past 60 years, Qatar's system of health care has evolved massively.^13^ The first hospital in Qatar, Rumailah Hospital, was established in 1957 and it is currently part of the large public healthcare complex of the country.^14^ Currently, the State of Qatar is home to a large number of hospitals and clinics. Primary Health Care Centers are public medical facilities that are dispersed throughout the nation and offer primary healthcare services.^15^

Hamad Medical Corporation was first founded in 1979.^16^ The majority of Qatar's healthcare is administered by Hamad Medical Corporation (HMC), a nonprofit healthcare public provider. There are currently 14 hospitals^17^, the National Ambulance Service, and home and residential care services. It founded Qatar's first academic health system, fusing cutting-edge study, education, and clinical care. HMC's research environment has made Qatar one of the four finest Arabic countries in research paper publication.^18-19^ The State of Qatar's dialysis services is covered by the nephrology division of HMC. There are 7 dialysis units nationwide.^11-12^

## Methods

### Study population

**Figure 1.**
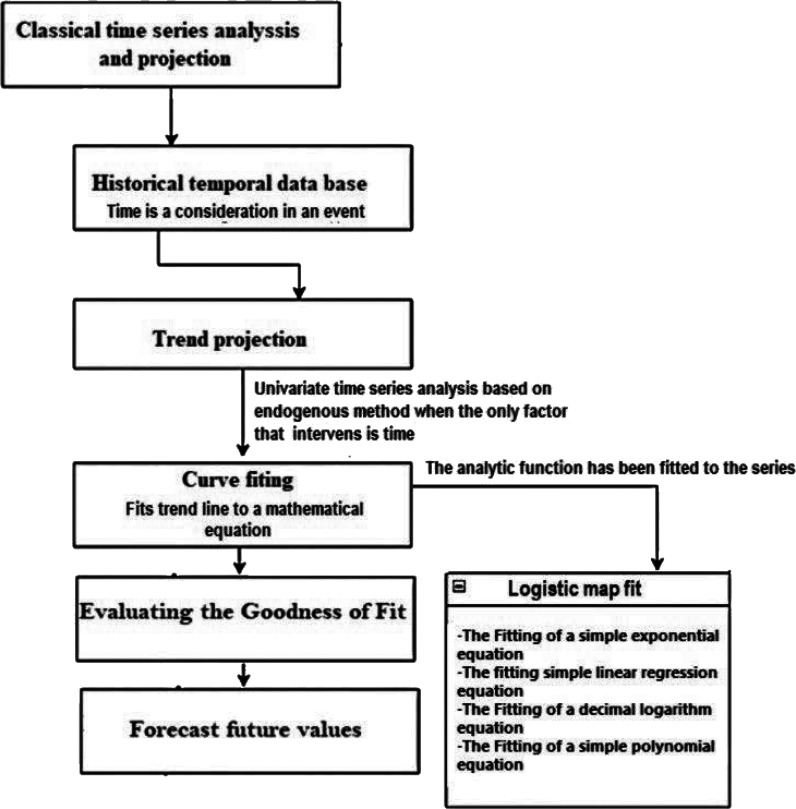
Schematic diagram of forecasting using a trend-based process time-series model. NOTE: Time-series analysis is a statistical method that examines trend data and time-series data.

In this research, we included all ESKD patients receiving HD at any of the HMC dialysis facilities between January 1, 2012, and December 31, 2021 (Figure 1). We incorporated ESKD patients on chronic dialysis therapy for more than 3 months. We exclude patients who were dialyzed for less than three months. There are 197 HD stations distributed among the seven ambulatory dialysis units in Qatar. By December 31, 2021, the crude prevalence of dialysis in adults in Qatar reached 981, equivalent to 697.24 per million population, a rise of 13.3% over the previous year.

**Figure 2.**
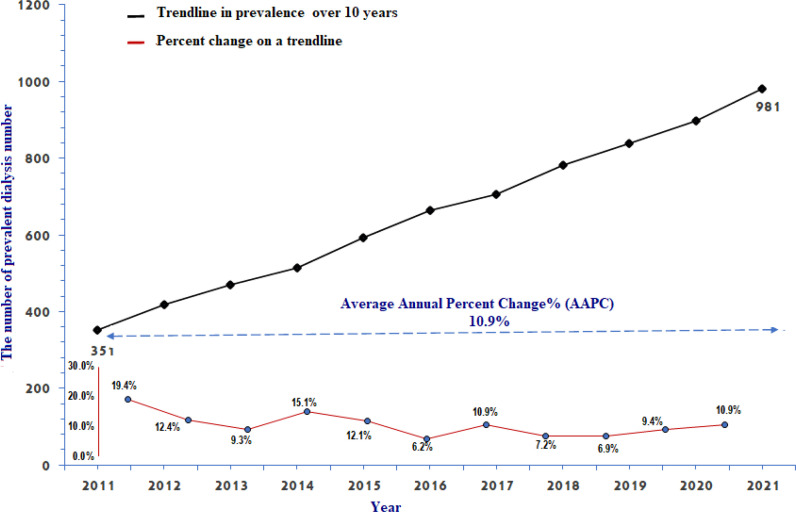
Shows the idea of change in such patients requiring dialysis over time. NOTE: • The time-series plot for the number of frequent dialysis patients during the study period is represented by the blue line • The red line indicates a simple annual percent change curve with distinct periods

The number of dialysis patients increased by an average annual percent change of 10.9% between 2012 and 2021. The annual percentage change (APC) varied, nevertheless, between 6.2% and 19.4%. APC stood at 10.9%, at the end of 2021 (Figure 2).

Although the FIFA World Cup preparations played a role in some of the population growth, we don't believe they had an impact on the expansion of the dialysis population in Qatar, as most expatriate workers and professionals are relatively young. According to the government, immigrants under the age of 40 make up more than 65% of Qatar's population.^20,21^ Additionally, all employees are required by law to before employment. However, local analysis of our data shows that this did not have an impact on the increase of the dialysis population growth. Our dialysis population consists of Qataris or people who live there permanently, most of whom are from Middle Eastern countries. Additionally, it is anticipated that population growth will be slow, steady, or plateau.^22^ We postulate that if population growth rates are sustained over a long period at the same pace, population fluctuations can be concealed.

### Annual prevalence of hemodialysis

The annual prevalence of patients necessitating HD was determined for 10 consecutive years, from 2012 to 2021. Data from this period was applied to construct an enhanced forecasting method for the next projection. Because it condenses historical trends, APC is frequently used to assess disease trends. In our study, this aids to track the growth in the number over time and demonstrates the difference in the form of an increase or decrease. We calculated it using the following formula:

**APC** = (Number Later–Number earlier)/| Number at an earlier time | × 100.

### Model identification

A time-series analysis (TSA) evaluates observations over time. Thus, the perception of an event depends on time to envisage future trends in disease, and only long-term trends can help determine hidden relationships used to forecast future trends in disease.^23^ Furthermore, depending on socioeconomic, environmental, and vector density variables, predictive models can predict future outbreaks. In epidemiological studies, forecasting is essential to track the spread of diseases over time. Also, a time-series is a sequence of data points tabulated (or listed or plotted) in a time-series {yt : t ∈ T};^1^ where T is the index set. A random function typically models a time-series, that is, a sequence of random variables. The time-series values in the forecasting system are known before time t, and the goal is to estimate y(t+h) using the available information at time t.^24^ Time-series research can be distinguished into three categories: descriptive modeling, TSA, and predictive modeling, commonly referred to as time-series forecasting. A diagram illustrating the process analysis for the predictive context and the statistical model applied in our investigation (Figure 1).

### Prediction models

TSA is the best method for using univariate temporal data among several conventional algorithms. Based on mathematical formulas and theorems^25,26^ that offer appropriate and usable representations of relationships, we standardized four health forecasting methods. Based on these justifications, we suggest utilizing TSA to estimate trends and predict the prevalence of ESKD necessitating dialysis in Qatar from 2022 to 2030 as follows:1) the simplest linear regression model, 2) the simplest exponential regression model, 3) logarithmic decimal regression model and 4) second-order polynomial regression model. The predicted data can facilitate the creation and execution of an overall strategic plan.

### Prediction performance evaluation metrics

The coefficient of determination (R^2^), mean absolute percentage error (MAPE), and maximum absolute deviation (MAD) was applied in assessing the expected prediction performance. Hence, our model was employed to predict the number of patients requiring dialysis until 2030 with a low prediction error.

The model, however, was able to match the data, as demonstrated by the coefficient of determination. In addition, MAPE was calculated to assess the accuracy of the prediction, because it is an easy-to-understand metric. To test for predictive variability, the MAD approach was used to compare individual observations. Therefore, this calculates the average absolute deviation of observations from their predictions. This demonstrates that the data points deviate from the mean. Finally, the statistics were computed using the following formula:

The coefficient of determination is expressed by the following formula (F1):

R^2^ = n √(ΣXY)-(Σx) (Σy) ÷√ [nΣx^2^-(Σx) ^2^] [niΣy^2^ − (Σy) ^2^]

MAPE is expressed by the following formula (F2):

MAPE = 1÷N Σ | x̅ -Xi| ÷Xi × 100

MAD is expressed by the following formula (F3):

MAD = Σ |Xi- x̅ |÷N

Where X_i_ represents the annual patient dialysis incidence data, 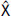 denotes the forecast data, x̅- symbolizes the mean of the yearly patient dialysis incidence data, N is the number of forecasts, and n is the number of data sets.

### Statistical analysis

All statistical analyses were conducted using Stata software (version 15.1, Stata Corp, College Station, TX, USA). A two-sided p-value of < .05 was considered statistically significant.

### Model fitting

Predictive modeling methods were generated based on the equations presented here.

The first model uses a simple linear regression function to make future predictions. It was applied to model the relationship between two variables using straight lines representing ordered pairs of data series (T, Y) where the data increases or decreases simultaneously over time to reduce the difference between predicted and actual values and has the following equation

Where ŷ(t) = b_1_t + b_0_.                             Eq (1)

Where b_0_ is the calculated slope and b is the calculated distance. Between the classes of linear and unbiased estimators, the ordinary least squares estimators b_0_ and b_1_ differ very little. Here “t” is the predictor, “ŷ” is the predicted variable, and ŷ is computed for each value of t if the data were linear.

Then, a simple exponential function is applied. As the data change, an exponential curve demonstrates processes that initially increase, accelerate quickly, drop, and finally slow down over time. It is described as a function with an exponent of one multiplied by the variable's exponent and a positive constant. While “k” and “b” are constants and (b, k) is determined using least squares, the equation below indicates the fit of the exponential model to the data

ŷ(t) = K e^bt^.                             Eq (2)

Additionally, “e” stands for “Euler's number,” and e = 2.718 demonstrates that a constant change in the independent variable “t” results in an exactly proportional change in the dependent variable (i.e., an increase or percentage decrease).

The base-_10_ logarithmic method, commonly referred to as the decimal logarithmic method, is the third technique we used, and it defines observations as rapid changes. The variables were then logarithmically transformed between independent variables “t” and dependent variables “ŷ.” The logarithmic function is denoted by the equation

ŷ(t) = log^10^t.                             Eq (3)

We can choose any base as long as it is positive but not equal to 1 when utilizing the change-of-base formula (log b (t)) or transforming a given logarithmic expression as a ratio or fraction of two logarithm operations. Thus, if we have a logarithm that uses a certain base, we can convert it to an equivalent ratio or fraction of two logarithmic operations and pick any base we want with “b” as a positive number equal to 1.

The nonlinear relationship between the dependent variable (y) and the independent variable (t) as a polynomial of degree n leads to the creation of a polynomial regression algorithm model. This holds for information that changes direction over time, such as upward or downward trends. The following equation indicates polynomial regression:

ŷ(t) = at^2^ + bt + c                             Eq (4)

Where t is a continuous value at instant t, and a, b and c are least squares values.

## Results

The four methods, associated trends, and mathematical equations used to predict the number of patients requiring dialysis in the future are illustrated by the experimental data (Figure 2). Based on data points collected throughout time, trends and equations were generated. Future prediction models have both strengths and weaknesses. The proposed model showed the number of patients who would require dialysis in the future using a time series.

**Figure 3.**
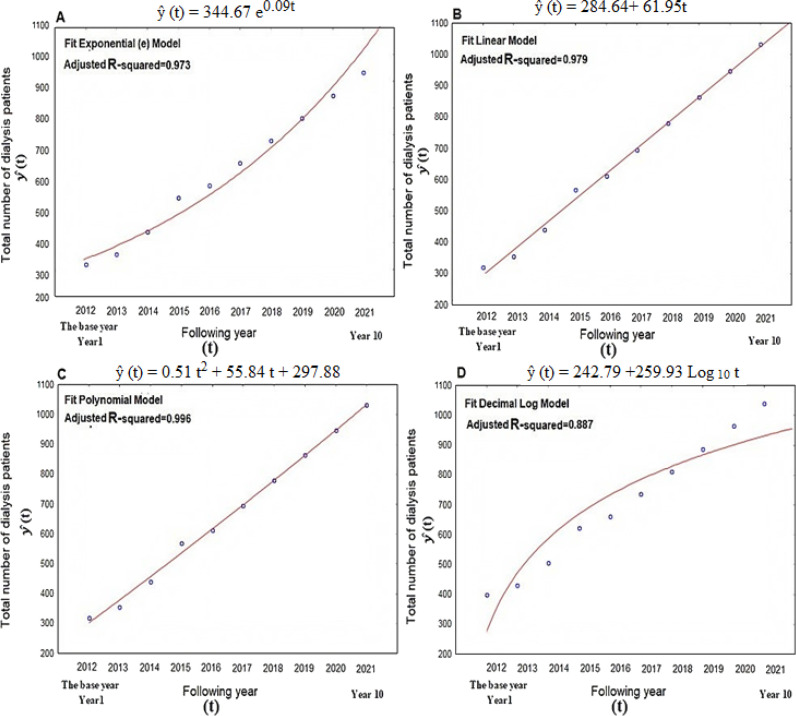
Different statistical models fitting. NOTE: P-value has been determined using the Chi-square test, and it was significant at p < 0.05 level.

The fit-criteria assessment plot (Figure 3) provides an overview of the four best scenarios that we evaluated between 2012 and 2021. The equations below demonstrate our strategy, which uses observations to gauge the likelihood and increase projections.

The first model is implemented using the simplest linear regression model illustrated by the equation: ŷ (t) = 284.64+61.95t.

The second model is executed using the simplest exponential regression model presented by the equation: ŷ (t) = 344.67 e^0.09t^.

The third model is implemented using a logarithmic decimal regression model shown by the equation: ŷ (t) = 242.79+259.93 Log _10_ t.

The fourth model is implemented employing a polynomial regression model of the second degree. It is denoted by the equation: ŷ (t) = 0.51t^2^ + 55.84t + 297.88.

We discovered a significant correlation between y (number of patients on dialysis) and t (years) (p < 0.05) for each of the four experimental models used to predict the prevalence of dialysis. Details of the corresponding fitting trajectory and mathematical equations are shown (Figure 3).

The best prediction method was chosen based on the accuracy assessment. Consequently, the polynomial model has a higher R^2^ value of about 0.996 compared to other evaluated models, indicating a good fit. The MAPE values ??for the log_10_ and polynomial models are 2.28% and 1.99%, respectively. Therefore, it is the most precise and trustworthy level II predictive model. The polynomial model demonstrated minimal variation with a MAD of 9.87%. With modest elasticity and minimal mistakes, the polynomial model of the current prediction so surpasses the other three forecasting models.

## Discussion

To our knowledge, this is the first study to estimate the prevalence of dialysis in Qatar using univariate time-series data. Over the following ten years, it is predicted that the State of Qatar will experience an increase in the number of dialysis patients; necessitating the construction of dialysis facilities (both in number and size). Demand forecasting provides data for future planning. This would make it easier for policymakers to predict the financial and logistical resources required for future implementation and policy recommendations.

Predictive analytics is a relatively simple concept in other fields, that accurately and reliably predicts the future of crucial health conditions based on diverse selection criteria influenced by multiple factors such as context, relevance, availability of historical data, forecast period, costs, and advantages of the organization, and the time available to prepare estimates.

Prediction accuracy is crucial for assessing a healthcare system's method. Four forecasting predictive models for the dialysis procedure were employed in this study. The original cohort included 2,600 patients enrolled in the Renal Transplant Treatment (RTT) program between January 2012 and December 2021. Only patients who received permanent dialysis were examined in this study. Based on ten years’ worth of historical data, trend estimates were obtained using forecasting and extrapolation methods.^27^ Our study shows that the difference in some points explains why annual prevalence rises while the growth rate decreases.^28^

With an R^2^ value of 0.996, the polynomial methodology outperformed the other approaches. The findings indicated a significant correlation between prevalence count and time. This denotes that the polynomial fit line contained 100% of the data point. Additionally, the forecasting outcomes of this polynomial model are listed (Table 2), where the absolute case numbers are summarized as the annual prevalence count.

Following polynomial projections, the prevalent dialysis cases are presumed to rise slightly during the projection period (2022–2030), with the markedly increasing prevalence considered realistic.

**Figure 4.**
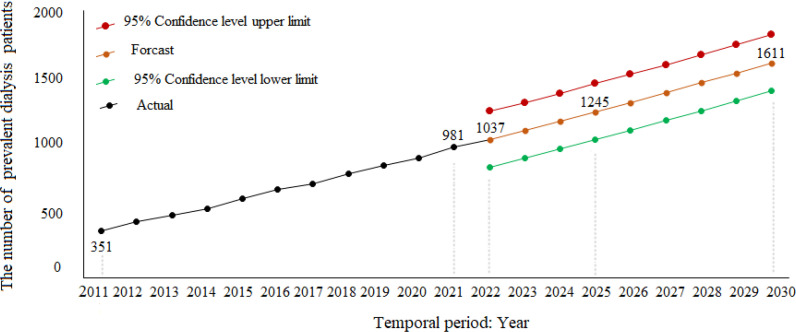
Forecasting and fitting polynomials to predict the number of dialysis patients in Qatar between 2022 and 2030.

According to the selected model, the expected number of dialysis patients in Qatar has increased (Figures 3 and 4). Additionally, the estimates of dialysis prevalence vary from year to year. Still, a general upward trend was noticed. It was 1037 (95% CI, 974–1126) in 2022, 1245 (95% CI, 911–1518) in 2025, and 1611 (95% CI, 1378–1954) in 2030. Therefore, we can observe a steady decrease in APC from 0.66 to 0.49 between 2022 and 2030. The APC in dialysis prevalence decreased, suggesting that the behavior of dialysis patients remained constant. We resolved that there is a mean increase of 70 new dialysis patients per year, with a maximum of 75 and a minimum of 64.

Following the results of this study, it is evident that the number of people who are anticipated to need dialysis will increase significantly shortly (Table 2). A prevalence estimate of 1037 patients in 2022 represents an increase of 56 cases (5.7%) from 2021, of which 78% had HD and only 22% had PD. The number of fatalities, however, is increasing annually. Therefore, it is recommended that the number of dialysis facilities be expanded in Qatar in accordance with the most recent standards to give adequate care to patients with end-stage renal disease.^29^ There is an urgent need for better governance, consolidation, standardization, and the creation of comprehensive plans to manage potential issues.

Understanding the growth rate of the ESKD population requiring dialysis is critical for planning health services, boosting preventive health care/services, creating alerts for patient overflow management in peak health service demand situations, and substantially reducing associated consumables and personnel costs. Hemodialysis and PD would cost US$88.16 billion globally in 2021. 2022 to 2030, this is anticipated to rise at an average annual rate of 6.61% from 2022 to 2030.^30^

Our study is the first to evaluate dialysis-predicted demand requirements using a straightforward approach supported by time-series trends during which only the passage of time intervenes. This method offers planning advice, and aids in estimating and assessing the development of dialysis using only the counting and evolution of the number of patients each year without the need for additional complex data (e.g., mortality, age, and comorbidities), that demand more time and sophisticated methods for health forecasting.

Other studies have scrutinized this increase.^31^ McCullough et al. reported on an open-ended fragmented simulation model to appraise the incidence and prevalence of ESKD within the United States by 2030.^32^ They demonstrated that the prevalence of ESKD is predicted to rise to 2700–3500 per million by 2030, an increase of 29–68% over the 2015 prevalence of 2087 per million. Recent findings indicate that the COVID-19 pandemic may have a conflicting impact on the expected growth of the ESKD population (i.e., increased mortality and a decline in transplantation).^33-36^

### Limitations

Our study has a few limitations. Due to our understanding that this group did not contribute to the expansion of the dialysis population, our forecast model did not consider unusual population growth that could be related to FIFA World Cup 2022 event. Another limitation is that our work focuses on finding the best mathematical model to forecast dialysis population growth although it also serves a therapeutic purpose, and it will be helpful to plan future resource requirements for dialysis patient care.

## Conclusions

The mathematical models presented in this work can accurately and simply anticipate the number of patients in Qatar who will eventually require dialysis. We discovered that the polynomial methodology outperformed the other methods. As a result, the conclusions drawn from this study's projections can serve as a starting point for discussing Qatar's ESKD program and its future challenges. This forecasting model predicts a rise in dialysis patients, necessitating the development of dialysis facilities (both in number and size).

### Abbreviations

Annual percentage change (APC)

Coefficient of determination (R^2^)

End-stage kidney disease (ESKD)

Hamad Medical Corporation (HMC)

Hemodialysis (HD)

Mean absolute deviation (MAD)

Mean absolute percentage error (MAPE)

Non-communicable diseases (NCDs)

Peritoneal dialysis (PD)

Renal Transplant Treatment (RTT)

Time-series analysis (TSA)

### Competing statement

We declare that none of the authors have to compete for financial or non-financial interests.

### Data availability

All data are fully available without restriction upon request.

### Financial disclosure

The author(s) received no specific funding for this work.

### Ethical approval

Our study was exempted from IRB as there were no human subjects or data included.

### Contribution statement

Abdullah Hamad and Mohamad Alkadi: significant contribution to the study design, literature review, interpretation of data, drafting the article and revising it critically, final approval of the version to be published, agreement to be accountable for all aspects of the work to ensure that any concerns about the accuracy or integrity of any part of the work are appropriately investigated and addressed. Hafedh Ghazali: substantial contribution to the study design, analysis of data, final approval of the version to be published, agreement to be held accountable for all facets of the work in order to make sure that concerns about the accuracy or integrity of any part of the work are appropriately examined and resolved. Anas Al Halabi2, Habas Elmukhtar 1: literature review, interpretation of data, drafting the article, final approval of the version to be published, agreement to be accountable for all aspects of the work in ensuring that questions associated with the accuracy or integrity of any part of the work are appropriately analyzed and resolved. Sahar M Ismail1, Hassan A. Al-Malki1: interpretation of data, drafting the article final approval of the version to be published, agreement to be accountable for all aspects of the work in ensuring that questions related to the accuracy or integrity of any part of the work are appropriately investigated and resolved. Mohamed BA1: substantial contribution to the study design, literature review, interpretation of data, drafting the article and revising it critically, final approval of the version to be published, agreement to be accountable for all aspects of the work in ensuring that questions related to the accuracy or integrity of any part of the work are appropriately investigated and resolved. All authors read and approved the final manuscript.

## Acknowledgments

We profusely thank; Ms. Ateya Heba; SN, Ms. Zitouni Youldez; Research assistant, Ms. Mathew Mincy; SN, and Ms. Ibrahim Rania; CNS.

## Figures and Tables

**Table 1:** Comparison of the value of R^2^ MAPE and MAD between proposed Models

Methods	Testing Power		
	R^2^ (%)	MAPE (%)	MAD
Exponential	97.3	5.39	34.14
Linear	97.9	2.62	11.85
Polynomial	99.6	2.28	9.87
The base 10 logarithm (The logarithm10)	88.7	1.99	10.07

Note**:** R^2^ = The coefficient of determination

MAPE = mean absolute percentage error

MAD = The mean absolute deviation

**Table 2:** Polynomial projections of people expected to require dialysis in Qatar between 2022 and 2030

Year	Projected value (Dialysis patients)	Estimated Annual PercentChange (APC) (%)
2022	1037	5.7
2023	1105	6.6
2024	1174	6.2
2025	1245	6.0
2026	1315	5.6
2027	1388	5.6
2028	1462	5.3
2029	1536	5.1
2030	1611	4.9

**NOTE:** Each year, there are between 200 and 250 new patients added to the dialysis community; however, there is only a net gain of about 60 to 70 patients as a result of patient losses brought on by transplantation, death, patient relocation abroad, and treatment termination.
